# microRNA-184 functions as tumor suppressor in renal cell carcinoma

**DOI:** 10.3892/etm.2015.2199

**Published:** 2015-01-21

**Authors:** ZHENGMING SU, DUQUN CHEN, YIFAN LI, ENPU ZHANG, ZUHU YU, TING CHEN, ZHIMAO JIANG, LIANGCHAO NI, SHANGQI YANG, YAOTING GUI, JIONGXIAN YE, YONGQING LAI

**Affiliations:** 1 Department of Urology, Peking University Shenzhen Hospital, Institute of Urology of Shanghai PKU-HKUST Medical Center, Shenzhen, Guangdong 518036, P.R. China; 2 Shantou University Medical College, Shantou, Guangdong 515041, P.R. China; 3 Guangdong and Shenzhen Key Laboratory of Male Reproductive Medicine and Genetics, Institute of Urology, Peking University Shenzhen Hospital, Shenzhen PKU-HKUST Medical Center, Shenzhen, Guangdong 518036, P.R. China; 4 Anhui Medical University, Hefei, Anhui 230032, P.R. China

**Keywords:** renal cell carcinoma, microRNA, miR-184

## Abstract

microRNAs (miRNAs) are evolutionarily conserved, endogenous, small, noncoding RNA molecules of approximately 22 nucleotides in length that function as post-transcriptional gene regulators. Their aberrant expression may be involved in human diseases, including cancer. Although miRNA-184 (miR-184) has been reported in other tumors, its function in renal cell carcinoma (RCC) is still unknown. The aim of the present study was to investigate the role of miR-184 in RCC. The impacts of miR-184 on cell migration, proliferation and apoptosis were evaluated using migration scratch, 3-(4,5-dimethylthiazol-2-yl)-2,5-diphenyltetrazolium bromide (MTT) and flow cytometry assay. Our studies revealed that miR-184 mimic significantly inhibits cell migration, suppresses cell proliferation and induces renal cancer cell apoptosis *in vitro* when compared with the negative control (P<0.05). In this study, it was observed that miR-184 played a significant role as a tumor suppressor in RCC. Therefore, miR-184 may be a promising therapeutic target for renal cancer treatment in the future.

## Introduction

Renal cell carcinoma (RCC) is the second most common genitourinary tumor, accounting for approximately 3% of all adult malignancies (1). Worldwide incidence and mortality rates are rising at a rate of approximately 2–3% every decade (2). Approximately 70% of patients present with localized diseases and the malignancy generally resists traditional oncological therapies including chemotherapy and radiotherapy. Radical or partial nephrectomy remains the mainstay of curative treatment (3). Although certain environmental and genetic factors have been identified as being associated with RCC, the molecular mechanisms involved in the initiation and progression of the disease are still unclear (4). In addition, more than one-third of patients may have metastasis when diagnosed, and half of patients may suffer recurrence even following nephrectomy (5). Therefore, an urgent need to identify new, sensitive, reliable biomarkers and develop new targeted therapies is emphasized for RCC.

Currently, one of the most prevalent and progressive approaches for the molecular characterization of tumors is based on microRNA (miRNA) expression profiles. miRNAs are endogenous noncoding 19–23 nucleotide RNAs involved in post-transcriptional regulation of gene expression (6), and play significant roles in a variety of biological processes, including proliferation, migration, differentiation and apoptosis (7). Mutated or abnormally expressed miRNAs have been identified as oncogenes or tumor suppressors in a number of human cancers, including RCC (8–10). It has been reported that miR-184 is widely dysregulated in various human cancers, including tongue squamous cell carcinoma (11), neuroblastoma (12), nasopharyngeal carcinoma (13) and hepatocellular carcinoma (HCC) (14), indicating that miR-184 may play a significant role in oncogenesis. Previous studies have demonstrated that miR-184 was downregulated in RCC, which may have potential significance in the occurrence and development of RCC (15). However, knowledge on the mechanism of action of miR-184 in RCC is limited. Therefore, there is a need to research this function.

The aim of our study was to examine the effects of miR-184 on proliferation, migration and apoptosis in two RCC cell lines and to lay the foundation for the further study of the pathogenesis of renal cell carcinoma.

## Materials and methods

### Cell culture

This study was approved by the institutional review board and ethics committee of Peking University Shenzhen Hospital, Shenzhen, China. Two human renal carcinoma cell lines were used, namely 786-o and ACHN, purchased from the American Type Culture Collection (ATCC, Manassas, VA, USA). The cell lines were incubated in Dulbecco’s modified Eagle’s medium (Invitrogen, Carlsbad, CA, USA) supplemented with 10% fetal bovine serum (Shanghai ExCell Biology, Shanghai, China) and 1% antibiotics (100 μg/ml penicillin and 100 mg/ml streptomycin sulfates) and maintained in a humidified incubator (5% CO_2_) at 37°C.

### Cell transfection efficiency

The miR-184 mimic or negative control was chemically synthesized by Shanghai GenePharma Co., Ltd. (Shanghai, China). The sequences were as follows: miR-184 mimic sense strand 5′-TGGACGGAGAACTGATAA GGGT-3′, and antisense strand 5′-CCTTATCAGTTCTCCGTC CATT-3′; miR-184 negative control sense strand 5′-TTCTCC GAACGTGTCACGTTT-3′, and antisense strand 5′-ACGTGA CACGTTCGGAGAATT-3′. ACHN and 786-o cells of 60–80% confluence were transfected with miR-184 mimic or negative control using Lipofectamine 2000 reagent (Invitrogen) according to the manufacturer’s instructions. Transfection efficiency and miR-184 expression changes were confirmed by fluorescence microscopy and reverse transcription-quantitative polymerase chain reaction (RT-qPCR). The culture medium was replaced by fresh medium 6 h after transfection, and the transfection efficiency was observed in cells which were transfected with green fluorescent protein Fam-labeled negative control (Shanghai GenePharma Co., Ltd.). The cells were harvested and total RNAs were extracted for RT-qPCR 24 h after transfection.

### RT-qPCR

Total RNA was extracted from cells using TRIzol reagent (Invitrogen) and purified with an RNeasy Maxi kit (Qiagen, Valencia, CA, USA) according to the manufacturer’s instructions. Only the RNA samples with 260/280 ratios of 1.8–2.0 were used for further investigation. A total of 1 μg total RNA was reverse transcribed into cDNA using the miScript reverse transcription kit (Qiagen) according to the manufacturer’s instructions. The RT-qPCR reaction of miR-184 was performed in a Lightcycler 480 real-time PCR system (Roche Diagnostics GmbH, Mannheim, Germany) using a miScript SYBR-Green PCR kit (Qiagen) according to the instructions and using U6 as an endogenous control. The 20 μl reaction mixture contained 10 μl 2X QuantiTect SYBR-Green PCR Master mix, 2 μl 10X miScript universal primer, 0.4 μl specific miRNA primer, 1 μl cDNA template and RNase-free water. The forward primer sequence of miR-184 was 5′-TGGACGGAGAACTGATAAGGGT-3′. Reverse primers were provided by the miScript SYBR-Green PCR kit. The forward primer of U6 was 5′-CTCGCTTCGGCAGCACA-3′ and the reverse primer was 5′-ACGCTTCACGAATTTG CGT-3′. PCRs were performed on cDNA of the mimic and negative control group in triplicate for each set. The protocol for PCR was 95°C for 15 min, followed by 40 cycles of 94°C for 15 sec, 55°C for 30 sec and 72°C for 30 sec. The miR-184 expression level was determined using the ΔΔCt method.

### Cell proliferation assay

The proliferation potential of cells was measured using the 3-(4,5-dimethylthiazol-2-yl)-2,5-diphenyltetrazolium bromide (MTT) assay. 786-o and ACHN cells were seeded into 96-well culture plates at a cell density of 6,000 cells/well in growth medium and transfected with 10 pmol miR-184 mimic or negative control. A blank control was set up with medium only. Cell growth was assayed by the addition of 20 μl MTT (5 mg/ml, Sigma, St. Louis, MO, USA) to each well, and the plate was incubated for 4 h at 37°C. The proliferation assay was performed for 3 days and cell growth was assayed at every 24 h interval. Then, the reaction was stopped by addition of 150 μl dimethyl sulfoxide (Sigma). After agitating for 15 min at room temperature, the optical density (OD) of each sample at the wavelength of 490 nm was measured with an enzyme immunoassay instrument (Bio-Rad, Hercules, CA, USA). Assays were repeated at least three times.

### Cell migration assay

Cell migration was examined by scratch assay according to the methods previously described (16). Approximately 500,000 cells (786-o and ACHN) were seeded in each six-well dish and transfected with miR-184 mimic (100 pmol) or negative control (100 pmol) 24 h later using Lipofectamine 2000. After 6 h of transfection, a sterile 200-μl pipette tip and markers were used to make a scratch in the cell monolayer. After scratching, the cells were washed with phosphate-buffered saline (PBS) medium three times and incubated at 37°C. Images of the scratches were acquired with a digital camera system at 0 and 24 h after the scratches were made at the same points. MIAS-2000 software (Leica Microsystems GmbH, Wetzlar, Germany) was used to determine the migration distance (μm). The experiments were performed in triplicate, and repeated at least three times.

### Cell apoptosis assay

The extent of apoptosis was evaluated using an Annexin V-fluorescein isothiocyanate (FITC)/propidium iodide (PI) detection kit (Invitrogen). 786-o and ACHN were cultured at 37°C and 5% CO_2_ in six-well plates and transfected with miR-184 mimic or negative control at a confluence of approximately 60%. For apoptosis assays, floating and adherent cells were collected 48 h after transfection and then combined and washed twice with pre-chilled PBS and resuspended in in 1X binding buffer (Invitrogen). In total, 5 μl Alexa Fluor^®^ 488 Annexin V (Invitrogen) and 3 μl PI (Invitrogen) were added to 500 μl cell suspension, and the samples were analyzed within 15 min of staining. The fluorescence was analyzed by flow cytometry (Beckman Coulter, Miami, FL, USA) using an excitation of 488 nm, according to the manufacturer’s instructions. Each experiment was performed at least three times.

### Statistical analysis

Statistical significance was determined with the t-test. Each experiment was repeated at least three times. The results were expressed as the means ± standard deviation. A two-tailed P<0.05 was considered to indicate a statistically significant difference. Statistical analysis was carried out with the SPSS 16.0 statistical software (SPSS, Inc., Chicago, IL, USA).

## Results

### Cell transfection efficiency

**Figure 1:**
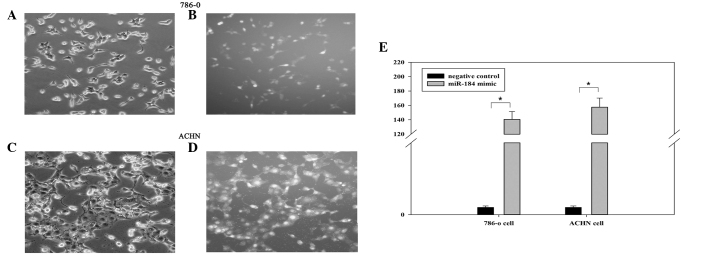
Transfection efficiency and miR-184 expression changes are confirmed by fluorescence microscopy and reverse transcription-polymerase chain reaction. (A and B) Images of 786-o cells transfected with Fam-labeled negative control 6 h after transfection in the same field; (C and D) Images of ACHN cells transfected with Fam-labeled negative control 6 h after transfection in the same field; (E) Fold changes of miR-184 in 786-o and ACHN cells 24 h after transfection. All experiments were performed in triplicate (^*^P<0.05).

To investigate the biological role of miR-184 in renal cancer, miR-184 mimic and negative control were transfected into renal cancer cell lines (786-o and ACHN). The Fam-labeled negative control was transfected into cells, and the transfection efficiency was analyzed by fluorescence microscopy 6 h after transfection. As shown in Fig. 1A–D, the transfection efficiency was ~85 and 90% in 786-o and ACHN cells, respectively. Furthermore, the fold changes of miR-184 determined by RT-qPCR assay in 786-o and ACHN cells were 140.67 and 157.57, respectively (Fig. 1E, P<0.05).

### miR-184 mimic inhibits renal cancer cell migration

**Figure 2:**
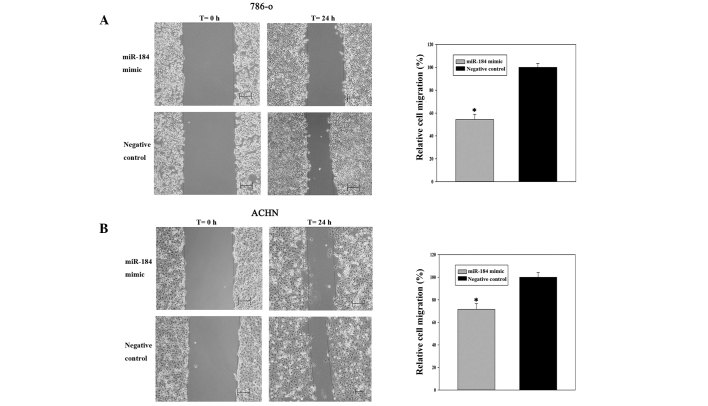
Effects of the miR-184 on cell migration in renal cell carcinoma cell lines. (A) miR-184 inhibited cell migration in 786-o cells; (B) miR-184 inhibited cell migration in ACHN cells. All experiments were performed in triplicate, and a representative image is shown (^*^P<0.05).

Scratch assays were performed to observe the function of miR-184 in cell migration. As shown in Fig. 2A and B, cell migration was significantly inhibited in the groups transfected with miR-184 compared with those in the negative control. The inhibition rates of migration were 45.58% for 786-o cells (P<0.05) and 28.44% for ACHN cells (P<0.05), indicating that miR-184 mimic inhibited the migration of renal cancer cells.

### miR-184 mimic suppresses renal cancer cell proliferation

**Figure 3:**
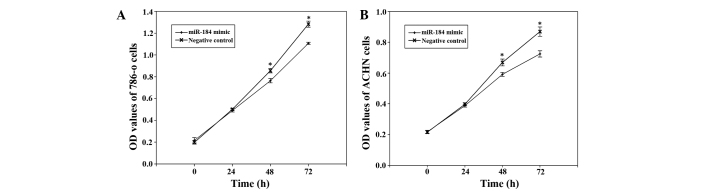
3-(4,5-dimethylthiazol-2-yl)-2,5-diphenyltetrazolium bromide assay for cell proliferation of 786-o and ACHN cells transfected with miR-184 mimic or negative control. (A) Cell proliferation of 786-o cells; (B) Cell proliferation of ACHN cells (^*^P<0.05).

To determine the potential role of miR-184 on the proliferation of renal cancer cells, MTT assays were performed. The miR-184 mimic group and negative control group were measured at 0, 24, 48 and 72 h after transfection. The OD values revealed that proliferation of 786-o cells was decreased by 2.3% (P>0.05), 10.75% (P<0.05) and 13.72% (P<0.05), while proliferation of ACHN cells was decreased by 2.4% (P>0.05), 11.57% (P<0.05) and 16.67% (P<0.05) at 24, 48 and 72 h after transfection, respectively, suggesting that miR-184 mimic inhibited the growth of 786-o and ACHN compared with the negative control (Fig. 3).

### miR-184 mimic induces renal cancer cell apoptosis

**Figure 4:**
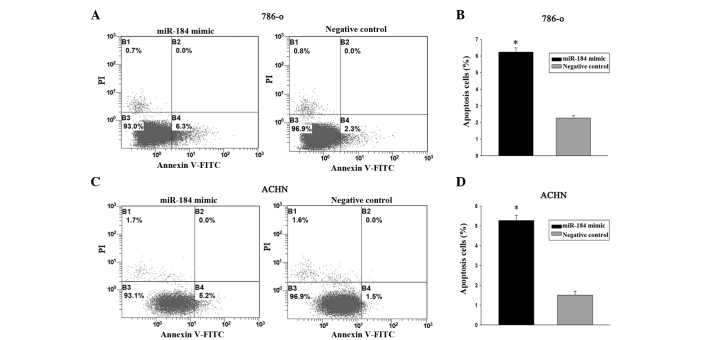
Effects of miR-184 on cell apoptosis in renal cell carcinoma cell lines. miR-145 induced cell apoptosis in 786-o cells (A) and ACHN cells (C). Cell apoptosis was measured by flow cytometry analysis with Annexin V-fluorescein isothiocyanate double-labeled. (B and D) Comparison of cell apoptosis rates following transfection with miR-184 mimic and negative control. The experiment was repeated three times. Data were the average of three independent experiments (^*^P<0.05).

The effects of miR-184 on cell apoptosis were further evaluated. 786-o and ACHN cells were transfected with miR-184 mimic and negative control for 48 h. Flow cytometry analysis demonstrated that the apoptosis rates of 786-o cells transfected with miR-184 mimic and control were 6.2% versus 2.2% (P<0.05) while the apoptosis rates of ACHN cells were 5.2% versus 1.5% (P<0.05). These data demonstrated that miR-184 mimic promoted renal cancer cell apoptosis (Fig. 4).

## Discussion

It is well known that RCC is one of the most common types of cancers affecting males and females. Despite the great advances in cancer therapy, major limitations still exist in managing RCC (17). Traditional chemotherapy and radiation are not effective in the treatment of advanced RCC. Therefore, searching for alternative treatment strategies remains a priority.

miRNAs are a group of endogenous, small, noncoding RNAs that have been observed in numerous organisms and are involved in post-transcriptional gene regulation through base pairing to partially complementary sites, notably in the untranslated region of mRNA (18). Using microarray technology, systematic expression analysis of miRNA profiles has been performed in several human tumors, including breast (19), lung (20) and renal cancer (21). Dysregulation of miRNAs has been observed between tumors and normal tissues and in distinct stages of cancer, indicating a possible correlation between miRNAs and oncogenesis. For example, miR-21, miR-451 and miR-145 have been identified as being associated with carcinogenesis and development of cancer by targeting oncogenes or anti-oncogenes (16,22,23).

In previous studies, miRNA expression profiles were demonstrated to be potential applications for the diagnosis, prognosis and treatment of tumors (24,25). These data are consistent with the hypothesis that miRNAs play an essential role in the development and progression of human cancers. Among these miRNAs, miR-184 is one of the most frequently studied in cancer biology.

There is evidence to suggest that aberrant expression of miRNAs in tumors contributes to human tumorigenesis by affecting the expression of multiple genes (26). miR-184 has been reported extensively in human cancers, suggesting that it may function as an oncogene in a variety of tumors. One comprehensive miRNA profiling of prostate cancer revealed that miR-184 was upregulated in high-grade tumors (27). Inhibition of miR-184 in tongue squamous cell carcinoma cells reduced cell proliferation and induced apoptosis (11). Furthermore, miR-184 is upregulated in human HCC cell lines and tissues, and it post-transcriptionally regulates SOX7 expression and promotes cell proliferation in HCC (14). Other scholars demonstrated that miR-184 has a tumor suppressive role in cancers. miR-184 inhibits neuroblastoma cell survival and promotes apoptosis by targeting AKT2 (12). Taken together, these studies indicate a possible role of miR-184 in modulating tumor progression. Our previous studies successfully identified numerous aberrant expressions of miRNAs in RCC by massively parallel sequencing technology, and revealed that miR-184 was significantly downregulated in RCC (10); then, researchers observed that the expression of miR-184 was downregulated in RCC tissues (15). However, the biological role of miR-184 in RCC is not fully elucidated, and several methods have been used to study their function.

In the present study, the functions of miR-184 on cell migration, proliferation and apoptosis were analyzed by transfecting miR-184 mimic and negative control into renal cancer 786-o and ACHN cells. Our results demonstrated that cells transfected with miR-184 mimic exhibited less cell migration, proliferation and more cell apoptosis compared with the negative control groups. The results provide new insight into the roles and possible mechanisms of miR-184 in the occurrence and development of RCC.

Based on the above, the results appear contradictory in that miR-184 was characterized as an oncogene in certain cancers and a tumor suppressor in others. This contradiction may be explained by the ‘imperfect complementarity’ of the interactions between miRNAs and target genes (28). Researchers observed that miRNAs post-transcriptionally regulated the expression of more than 30% of protein coding genes by translational repression, which also regulated the expression of several putative target genes by binding to a complementary sequence predominantly in their untranslated region. However, the bindings are not always completely complementary, particularly in mammals (29,30). Moreover, further research should be conducted to determine the roles and target genes of miR-184 in renal cell carcinoma.

In conclusion, our results revealed that miR-184 dramatically suppressed cell proliferation and migration and induced cell apoptosis in renal cancer cell lines and plays an significant role in RCC. In addition, our data suggest that miR-184 may be a promising therapeutic target for renal cancer treatment in the future. Further research is still needed to explore the roles and target genes of miR-184.
